# Inflammation and Resolution in Obesity-Related Cardiovascular Disease

**DOI:** 10.3390/ijms27010535

**Published:** 2026-01-05

**Authors:** Paschalis Karakasis, Panagiotis Stachteas, Panagiotis Iliakis, Georgios Sidiropoulos, Konstantinos Grigoriou, Dimitrios Patoulias, Antonios P. Antoniadis, Nikolaos Fragakis

**Affiliations:** 1 Second Department of Cardiology, Hippokration General Hospital, Aristotle University of Thessaloniki, Konstantinoupoleos 49, 54642 Thessaloniki, Greeceaantoniadis@gmail.com (A.P.A.); fragakis.nikos@gmail.com (N.F.); 2 First Cardiology Department, School of Medicine, Hippokration General Hospital, National and Kapodistrian University of Athens, 10679 Athens, Greece; panayiotisiliakis@gmail.com; 3 Department of Cardiology, Georgios Papanikolaou General Hospital, Leoforos Papanikolaou, 57010 Thessaloniki, Greece; sidiropoulos.georges@gmail.com; 4 Department of Pharmacology, University of Athens, 75 Mikras Asias Avenue, 11527 Goudi, Greece; dinosgrigoriou@gmail.com; 5 Second Propedeutic Department of Internal Medicine, Faculty of Medicine, School of Health Sciences Aristotle, University of Thessaloniki, 54642 Thessaloniki, Greece; dipatoulias@gmail.com

**Keywords:** obesity, inflammation, resolution pharmacology, heart failure, atrial fibrillation, coronary artery disease, adipokines

## Abstract

Obesity-associated inflammation underlies much of cardiometabolic pathology, reflecting the convergence of chronic, low-grade systemic immune activation with region-specific maladaptation of adipose depots. Among these, epicardial adipose tissue (EAT)—a visceral fat layer contiguous with the myocardium and sharing its microvasculature—functions as a cardio-proximal immunometabolic interface that influences atrial fibrillation, heart failure with preserved ejection fraction, and coronary atherogenesis through paracrine crosstalk. These relationships extend beyond crude measures of adiposity, emphasizing the primacy of local inflammatory signaling, adipokine flux, and fibro-inflammatory remodeling at the EAT–myocardium interface. Of importance, substantial weight reduction only partially reverses obesity-imprinted transcriptional and epigenetic programs across subcutaneous, visceral, and epicardial depots, supporting the concept of an enduring adipose memory that sustains cardiovascular (CV) risk despite metabolic improvement. Accordingly, therapeutic strategies should move beyond weight-centric management toward mechanism-guided interventions. Resolution pharmacology—leveraging specialized pro-resolving mediators and their cognate G-protein-coupled receptors—offers a biologically plausible means to terminate inflammation and reprogram immune–stromal interactions within adipose and CV tissues. Although preclinical studies report favorable effects on vascular remodeling, myocardial injury, and arrhythmic vulnerability, clinical translation is constrained by pharmacokinetic liabilities of native mediators and by incomplete validation of biomarkers for target engagement. This review integrates mechanistic, depot-resolved, and therapeutic evidence to inform the design of next-generation anti-inflammatory strategies for obesity-related CV disease.

## 1. Introduction

Inflammation is fundamentally protective, containing and eliminating threats to the host [1]. Yet when termination fails or is delayed, an initially adaptive program hardens into chronic, low-grade activation that propagates tissue injury and metabolic disease [2]. In obesity—and in individuals who have surpassed their physiological lipid-storage capacity—systemic smoldering inflammation couples with a more aggressive, depot-centered response in adipose tissue, driving insulin resistance (IR) and maladaptive crosstalk with the liver, vasculature, and heart [3,4]. Against this backdrop, therapeutics that blunt upstream cytokines (for example, IL-1β, TNF, IL-6) have yielded, at best, modest metabolic benefit and carry nontrivial risks, including infection, limiting their routine use [5,6,7].

A complementary strategy is to engage the body’s endogenous “off-switches” for inflammation: the active resolution pathways [8]. Specialized pro-resolving mediators (SPMs)—lipoxins, resolvins, protectins, and maresins—act in a non-phlogistic manner to limit leukocyte influx, promote efferocytosis, and restore tissue homeostasis, including insulin signaling within adipose tissue [9,10]. Although clinical translation remains nascent, early evidence suggests that pro-resolution pharmacology can reprogram immune–stromal interactions without broad immunosuppression, positioning SPM pathways as mechanistically attractive candidates for cardiometabolic disease (CMD) modification [11].

Within this conceptual framework, this review provides a focused synthesis of how obesity-associated inflammation and impaired resolution translate into vascular, myocardial, and arrhythmic pathology, with particular emphasis on depot-specific adipose–cardiac crosstalk. By integrating mechanistic, translational, and clinical evidence and highlighting specialized pro-resolving mediators and their receptors as actionable nodes, it outlines how targeting resolution pathways in obesity may recalibrate cardiovascular (CV) risk and guide the development of future mechanism-based therapeutic strategies.

## 2. Scope and Literature Search

A literature search was performed in MEDLINE (PubMed) and Scopus, covering each database from its inception through November 2025, without language restrictions. Search terms combined free-text keywords and Medical Subject Headings related to obesity, distinct adipose tissue depots, inflammatory and pro-resolving pathways, and major cardiovascular conditions (atherosclerosis, endothelial dysfunction, cardiomyopathy, arrhythmias, and heart failure). Priority was given to mechanistic, translational, and clinical studies that directly examined the relationship between adipose-tissue biology and cardiovascular outcomes. Additional articles were identified by screening reference lists and using citation tracking. The overall search and study selection process is summarized in Supplementary Figure S1.

## 3. Inflammatory Pathways Driving Cardiometabolic Pathology in Obesity

**Figure 1:**
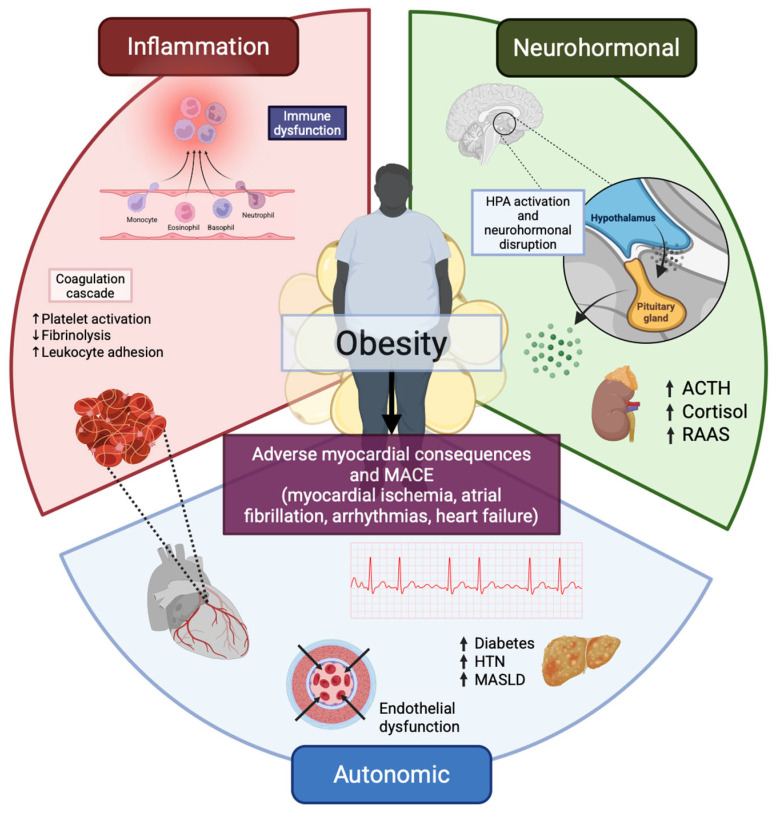
Inflammatory, neurohormonal, and autonomic pathways linking obesity with cardiometabolic disease. Obesity-related adipose tissue expansion leads to adipocyte hypertrophy and hypoxia, triggering HIF-1α activation, oxidative and ER stress, cell death, and fibrotic remodeling with impaired adipose expandability. These changes promote chemokine-driven immune cell infiltration, a shift toward pro-inflammatory M1-like macrophages and Th1/Th17 responses, NLRP3 inflammasome activation, and excess production of TNF-α, IL-6, IL-1β, IL-18, alongside an adipokine imbalance (↑ leptin/resistin, ↓ adiponectin). Spillover of cytokines, adipokines, and acute-phase reactants into the circulation sustains low-grade systemic inflammation, drives insulin resistance in the liver, muscle, and myocardium, and contributes to an atherogenic lipid profile. Downstream, endothelial dysfunction, oxidative stress, and vascular inflammation accelerate atherogenesis, while myocardial inflammation and fibrosis foster LV hypertrophy, HFpEF, and an AF-prone atrial substrate, further amplified by EAT/PVAT-derived paracrine signals. Hepatic steatosis/Metabolic Dysfunction-Associated Steatohepatitis (MASH) and renal injury act as additional inflammatory and neurohormonal amplifiers, in parallel with RAAS/SNS activation and a pro-thrombotic state, all compounded by impaired resolution of inflammation. ACTH, adrenocorticotropic hormone; HPA, hypothalamic–pituitary–adrenal (axis); HTN, hypertension; MACE, major adverse cardiovascular events; MASLD, metabolic dysfunction—associated steatotic liver disease; RAAS, renin–angiotensin–aldosterone system.

Inflammation is a central driver of obesity-related cardiometabolic pathology (Figure 1) [1,12]. Robust evidence—including multiple meta-analyses—demonstrates that circulating inflammatory biomarkers, notably C-reactive protein (CRP), independently predict CV events in both men and women. In the obese state, two interlocking inflammatory programs propagate disease: (i) a systemic, low-grade inflammatory milieu that injures the vascular endothelium and promotes multiorgan dysfunction; and (ii) a locally amplified adipose tissue inflammation that fosters peripheral IR and further augments systemic inflammation via cytokine and adipokine release [2,13]. Importantly, excess adiposity per se is not a prerequisite for these responses: individuals without overt obesity who have exceeded their physiological lipid-storage capacity can manifest both systemic and adipose-tissue inflammation, underscoring the primacy of lipid spillover and ectopic deposition over body mass alone [14,15].

Obesity-associated systemic low-grade inflammation arises through multiple, intersecting pathways. A leading framework posits that metabolic endotoxemia—the translocation of bacterial lipopolysaccharide (LPS) into the bloodstream—activates innate immune signaling cascades that precipitate peripheral insulin resistance, hyperglycemia, and end-organ injury, including metabolic dysfunction—associated steatotic liver disease (formerly non-alcoholic fatty liver disease) [16]. Potential extraintestinal and intestinal sources of LPS have been implicated. Oral dysbiosis with periodontal bleeding provides one conduit and has been linked to heightened CV risk [17]. Within the intestine, alterations in microbial composition together with diminished expression of epithelial junctional proteins erode barrier function, permitting lipopolysaccharide (LPS) to translocate into the circulation—often referred to, albeit informally, as “leaky gut” [18]. The downstream inflammatory load can be further magnified by defects in hepatic handling of LPS: impaired metabolic processing and reduced biliary excretion increase systemic exposure, while LPS simultaneously stimulates tumor necrosis factor (TNF) production by Kupffer cells, thereby exacerbating hepatocellular injury [19]. Beyond LPS-mediated pathways, ectopic lipid accumulation within the liver independently sustains low-grade inflammation and metabolic derangement [20]. Moreover, physical inactivity removes an important physiological brake on inflammation, insofar as contracting skeletal muscle ordinarily releases cytokines and myokines with anti-inflammatory and immunomodulatory properties; when muscle contractile activity is curtailed, this counter-regulatory influence wanes, biasing the system toward persistent inflammatory signaling [20].

Obesity-related disturbances of the gut microbiota are now thought to contribute to cardiovascular disease through mechanisms that extend beyond classical LPS-driven inflammation. In individuals with obesity, dietary patterns that promote dysbiosis, together with impaired intestinal barrier integrity, can lead to intermittent translocation of microbial products into the circulation and to broader changes in the circulating metabolome [21]. Among the metabolites that have been most consistently implicated are trimethylamine N-oxide (TMAO), short-chain fatty acids (SCFAs), and secondary bile acids [22]. TMAO is generated when intestinal microbes metabolize choline- and carnitine-containing nutrients to trimethylamine, which is then oxidized in the liver; higher TMAO levels have been associated with endothelial activation, disturbances in cholesterol handling, and increased platelet reactivity, a triad that is compatible with an enhanced atherothrombotic risk profile. By contrast, SCFAs such as acetate, propionate, and butyrate, produced through microbial fermentation of dietary fibre, signal through host G-protein-coupled receptors and related pathways to dampen inflammatory tone, promote regulatory immune responses, and modulate blood pressure regulation [23]. In parallel, bacterial conversion of primary into secondary bile acids can influence cardiometabolic risk via FXR- and TGR5-dependent effects on lipid trafficking, glucose homeostasis, and vascular inflammatory signalling [24]. Taken together, these lines of evidence support a mechanistic link between gut dysbiosis and obesity-associated cardiovascular disease and provide a rationale for therapeutic approaches that aim to preserve barrier function and restore a more favourable, cardioprotective metabolite milieu [25].

Evidence from clinical trials suggests that microbiota-targeted therapies may modestly modify cardiovascular risk profiles in patients with obesity, although data on hard outcomes are still limited. In overweight and obese adults, several randomized trials and meta-analyses report that multi-strain probiotic supplementation can lead to small reductions in body weight, BMI, LDL cholesterol, and, in some analyses, systolic blood pressure and circulating inflammatory markers compared with placebo, typically in the range of ~1 kg weight loss and a 3–4 mmHg fall in systolic blood pressure [26,27]. Prebiotic fibers and synbiotic combinations show broadly similar patterns, with improvements in fasting glucose, triglycerides, and waist circumference in cohorts with metabolic syndrome or coronary artery disease, although between-study heterogeneity, short intervention periods, and variable strain selection temper the strength of inference [28]. Conversely, pediatric trials and some recent adult studies in obesity and hypertension have yielded largely neutral results on composite cardiometabolic risk scores [29]. Most importantly, very few trials are powered for major adverse cardiovascular events, and systematic reviews therefore view probiotic and prebiotic approaches as promising adjuncts for risk-factor optimization rather than established cardioprotective therapies [30]. Larger, longer-term studies in patients with obesity and manifest CVD are needed to clarify whether these modest effects on intermediate phenotypes translate into meaningful reductions in clinical events.

The primary drivers of obesity-associated adipose inflammation remain incompletely defined, but the process is plausibly amplified by metabolic endotoxemia and systemic low-grade inflammation [31]. Chronic nutrient excess imposes sustained metabolic stress that tonically engages the NLRP3 inflammasome, increasing interleukin-1β (IL-1β) production. Although postprandial IL-1β can be physiologically supportive of insulin secretion and glycemic control, its persistent elevation in obesity shifts from adaptive to maladaptive, contributing to β-cell secretory exhaustion and dysglycemia [32].

Convergent tissue-level stressors further entrench inflammation. Adipocyte hypertrophy precipitates local hypoxia, while inadequate extracellular matrix (ECM) remodeling and emerging fibrosis impose mechanical strain that perpetuates sterile inflammatory signaling [33]. Obesity is also associated with immune and metabolic derangements—notably depletion of regulatory T (Treg) cells, disordered phosphocreatine energetics, endothelial dysfunction, and hyperinsulinemia-induced adipocyte senescence—each of which accentuates adipose inflammatory tone and IR relative to normal-weight states [34,35]. Finally, when adipocytes are overloaded with lipid, they undergo necrotic death, releasing danger signals that recruit and activate innate immune cells, thereby amplifying local inflammation and propagating systemic metabolic injury [36].

Irrespective of the initiating insult, inflamed adipose tissue shares several convergent features: heightened reactive oxygen species (ROS) burden; a reprogrammed adipokine–cytokine milieu characterized by increased leptin, visfatin, interleukin-6 (IL-6), and monocyte chemoattractant protein-1 (MCP-1/CCL2), alongside reduced adiponectin relative to non-inflamed depots; and augmented leukocyte recruitment with a pro-inflammatory bias [37,38]. Within this immune influx, T cells exhibit exhaustion phenotypes under chronic antigenic stimulation, whereas B cells accumulate, promote insulin resistance, and generate pathogenic antibodies [39]. In parallel, adipose macrophages adopt an M1-like program [40] and secrete tumor necrosis factor (TNF), thereby inhibiting canonical insulin signaling pathways in adipocytes [40]. The sustained, bidirectional crosstalk among adipocytes, macrophages, and T cells—amplified by redox stress and chemokine gradients—establishes a self-reinforcing feed-forward loop that perpetuates adipose inflammation and drives systemic metabolic dysfunction.

## 4. Activation and Resolution of Inflammation

Inflammation is a core physiological defense that continuously monitors and preserves tissue homeostasis [41]. When this program becomes dysregulated or persists beyond its intended window, it evolves into a chronic state that drives tissue injury and disease [12]. The preservation of organismal integrity therefore depends on an inflammatory response that is tightly calibrated and intrinsically self-limiting.

Of importance, the acute response is initiated when tissue sentinels recognize pathogen- or damage-associated molecular patterns through pattern-recognition receptors [42]. Downstream cytokine release induces vasodilation, increases vascular permeability, and orchestrates leukocyte trafficking [42]. Neutrophils are recruited first to phagocytose microbes and debris; monocytes arrive subsequently and differentiate into macrophages that contribute to resolution, in part through efferocytosis of apoptotic neutrophils. Depending on the nature of the insult—viral infection being a prototypical example—lymphocytes supply antibody production, cytokine signaling, and coordination of effector functions [43].

Under physiological conditions, this acute phase transitions to an active resolution program rather than a passive decay of signals [44]. Specialized SPMs—lipoxins, resolvins, protectins, and maresins—temper further leukocyte influx, enhance efferocytosis, and re-establish barrier integrity. Protein and gaseous pathways act in concert: IL-10 and TGF-β enforce anti-inflammatory checkpoints; annexin A1 accelerates neutrophil clearance and macrophage reprogramming; endogenous autacoids such as nitric oxide, hydrogen sulfide, and carbon monoxide modulate vascular tone, survival pathways, and pro-resolution signaling [45].

SPM biosynthesis from PUFA substrates is triggered at the height of inflammation [46]. Through engagement of G-protein-coupled receptors [47,48], SPMs execute a multifaceted resolution program that restricts additional leukocyte recruitment, limits collateral tissue injury, and restores homeostasis. In phagocytes, SPMs suppress continued neutrophil influx while favoring the recruitment of pro-resolving monocytes; in macrophages, they skew polarization toward an M2-like phenotype and augment efferocytosis, enabling non-phlogistic clearance of cellular debris. SPMs also establish feed-forward circuits that increase leukocyte production of pro-resolving lipids, thereby reinforcing resolution [49], and they transcriptionally repress pro-inflammatory programs—including TNF—curbing excessive signaling and tissue damage.

**Figure 2:**
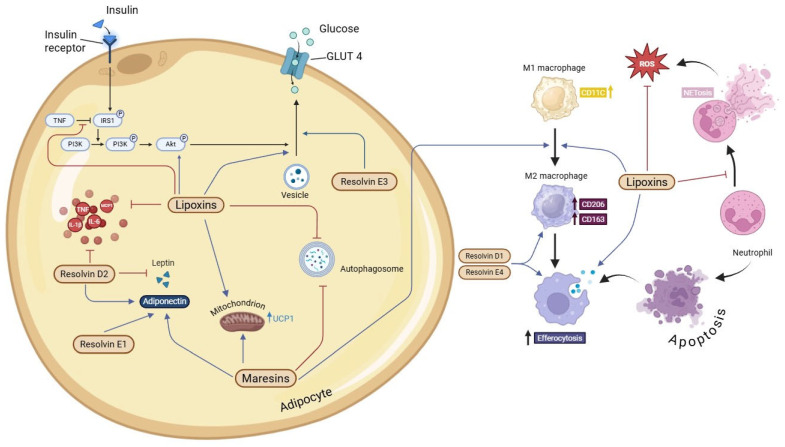
Pro-resolving therapeutic strategies to attenuate obesity-related inflammation and downstream disease. This figure summarizes interventions that activate endogenous resolution programs—principally the administration of specialized pro-resolving mediators (SPMs: lipoxins, resolvins, maresins)—to dampen adipose tissue inflammation in obesity. Across preclinical models, SPMs mitigate adipose and systemic inflammatory injury and limit organ damage; concordant human ex vivo data in adipose explants and cultured adipocytes demonstrate analogous anti-inflammatory and insulin-sensitizing effects. Mechanistically, tumour necrosis factor (TNF)—induced desensitization of insulin signalling is counteracted by SPMs: lipoxins restore insulin receptor substrate-1 (IRS1) phosphorylation and enhance Akt activation, thereby promoting GLUT4 vesicle translocation to the plasma membrane. SPMs also remodel the adipokine milieu—raising adiponectin, lowering leptin—and suppress pro-inflammatory cytokine production; maresins further drive adipocyte browning, reflected by increased uncoupling protein-1 (UCP1). At the leukocyte level, SPMs shift macrophages toward an anti-inflammatory, reparative (M2-like) phenotype, enhance efferocytosis of apoptotic neutrophils, and curb neutrophil extracellular trap formation (NETosis) and excessive reactive oxygen species (ROS) generation. SPM actions on cellular quality-control pathways should be interpreted as normalization of dysregulated autophagy/lysosomal flux under metabolic stress rather than inhibition of physiological autophagy.

Of note, these pathways converge on canonical anti-inflammatory and tissue-repair mechanisms (e.g., IL-10, TGFβ, annexin A1) that are directly relevant to metabolic inflammation. In adipose tissue, SPMs counter obesity-associated TNF signaling that desensitizes insulin pathways in adipocytes: lipoxins restore IRS-1 phosphorylation and stimulate Akt activation, facilitating GLUT4 vesicular translocation and improving insulin responsiveness [50,51,52]. As summarized in Figure 2 SPMs thereby reshape adipocyte function—normalizing insulin signaling, promoting a more insulin-sensitizing and anti-inflammatory adipokine profile, and, in the case of maresins, fostering adipocyte browning [53]. In parallel, SPMs reprogram leukocytes within adipose tissue by shifting macrophages toward reparative phenotypes, enhancing efferocytosis, and modulating T-cell and B-cell responses: TH1/TH17 effector activity is tempered, Treg function is augmented [54], and B-cell antibody responses are differentially regulated in a mediator- and context-dependent manner [55,56,57,58]. Vascular and interstitial dynamics are likewise targeted, with SPMs tightening endothelial junctions, restoring vascular integrity, and stimulating lymphatic clearance to resolve edema [59]. When this resolution circuitry is disrupted, inflammation becomes self-sustaining and chronic—a hallmark of several pathologies, including obesity [60].

## 5. Depot-Specific Immunometabolism

Human adipose depots display discrete immunometabolic programs with direct clinical implications [61]. Subcutaneous adipose tissue (SAT) is comparatively insulin-sensitive and angiogenic, whereas visceral adipose tissue (VAT) is enriched for stress-responsive and pro-inflammatory pathways that align with systemic IR and heightened cardiometabolic risk. Epicardial adipose tissue (EAT) is distinct among visceral depots: contiguous with the myocardium and sharing its microcirculation, it forms a cardio-proximal, inflammatory–lipotoxic niche capable of paracrine crosstalk with atrial and ventricular myocardium [62]. Contemporary syntheses indicate that EAT remodeling—marked by macrophage activation, adipokine signaling, and progressive fibrotic change—likely contributes to diastolic dysfunction and cardiac structural remodeling characteristic of heart failure with preserved ejection fraction (HFpEF), thereby extending causal pathways beyond general adiposity to depot-specific biology [62].

Multiple clinical and translational reports now converge on EAT as a disease-modifying substrate across atrial fibrillation (AF) [63,64], heart failure with HFpEF, and coronary artery disease (CAD) [65,66,67,68]. Invasive-hemodynamic cohorts demonstrate that higher EAT burden associates with impaired functional capacity and abnormal exercise hemodynamics, aligning with an immunometabolic role for EAT in limiting diastolic reserve [69]. Imaging and observational studies further connect EAT volume/attenuation with AF propensity—consistent with fibro-inflammatory signaling at the EAT–atrium interface—and suggest prognostic value across heart-failure populations [65,66,70,71].

Extending this paradigm to atherosclerosis, human tissue and serum analyses demonstrate CAD-linked perturbations in the EAT immune–adipokine axis—increased CD11c/CD206 macrophage polarization ratio, higher resistin, and lower apelin expression—even when crude depot size metrics do not differ, underscoring that qualitative inflammatory remodeling rather than depot quantity may be the salient CV signal [72]. Consistent with its central role in cardiometabolic disease, EAT in patients with CAD, obesity, or type 2 diabetes exhibits profound qualitative remodeling that extends beyond simple increases in depot volume [73]. Histological and molecular analyses indicate a shift from a more brown-like, metabolically active, and vasculoprotective phenotype toward a whitened, pro-inflammatory, and fibrotic profile, characterized by reduced mitochondrial and thermogenic signatures, impaired angiogenic capacity, and activation of stress- and danger-sensing pathways [74,75,76]. EAT from cardiometabolic patients shows increased production of inflammatory cytokines and chemokines (e.g., TNF-α, IL-6, IL-1β, MCP-1), an adverse adipokine milieu with higher leptin and resistin and lower adiponectin and apelin, as well as enrichment of pro-inflammatory immune cell subsets, including classically activated macrophages and activated T cells [77,78,79,80]. Notably, these qualitative immunometabolic alterations may be present even when crude EAT thickness or volume does not differ substantially between individuals with and without CAD, underscoring that inflammatory activation and secretory reprogramming—rather than depot size per se—likely represent the most clinically relevant CV signal [81,82]. Taken together, these data position EAT as both a biomarker and therapeutic target at the intersection of metabolism, electrophysiology, hemodynamics, and coronary atherogenesis.

Pharmacologic interventions that ameliorate systemic metabolic dysfunction can also modulate EAT quantity and phenotype, promoting a less inflammatory, more cardioprotective profile. Emerging clinical data demonstrate reductions in EAT with glucagon-like peptide-1 receptor agonists (GLP-1RAs), a finding that is mechanistically plausible given reported depot-level GLP-1 signaling and the substantial weight loss these agents induce; moreover, phenotype-specific responses across heart-failure syndromes have been proposed [83]. Activation of peroxisome proliferator-activated receptor-γ (PPARγ) has likewise been associated with decreases in EAT area and improvements in diastolic parameters despite overall weight gain, consistent with anti-inflammatory depot redistribution [84,85]. Notably, sodium–glucose cotransporter-2 (SGLT2) inhibitors, which have established pleiotropic effects, show particularly consistent effects: a recent network meta-analysis reported greater EAT reduction with SGLT2 inhibitors than with GLP-1RAs or structured exercise in individuals with type 2 diabetes and/or obesity, and a complementary meta-analysis linked SGLT2 therapy to lower EAT volume/thickness, providing a mechanistic bridge to observed benefits in heart-failure populations. Collectively, these observations justify the incorporation of EAT-focused imaging and functional end points in forthcoming cardiometabolic trials.

Perivascular adipose tissue (PVAT) represents a second, strategically located adipose niche with clear relevance to obesity-associated vascular pathology. In the healthy state, PVAT confers an anti-contractile and vasculoprotective influence on the underlying vessel wall by releasing a range of adipocyte-derived relaxing factors—among them adiponectin, hydrogen sulphide, nitric oxide–related mediators, and angiotensin 1–7—which collectively help preserve endothelial function, attenuate oxidative stress, and restrain vascular smooth muscle cell proliferation and migration [86]. With the development of obesity and broader metabolic derangement, this equilibrium is progressively lost. PVAT becomes hypertrophic and hypoxic and undergoes qualitative inflammatory reprogramming, characterized by attenuation of its anticontractile properties and the emergence of a pro-contractile, vasculotoxic secretome [87]. This remodeled PVAT is enriched in reactive oxygen species, pro-inflammatory cytokines and chemokines such as TNF-α, IL-6, and MCP-1, vasoactive mediators including angiotensin II and catecholamines, and maladaptive adipokines such as leptin, resistin, and chemerin [87]. In combination, these changes foster endothelial dysfunction, adventitial inflammation, neointimal hyperplasia, and more rapid atherosclerotic plaque development in the adjacent vasculature. Evidence from experimental models and clinical imaging now converges to implicate PVAT inflammation and phenotypic “whitening” in coronary and aortic plaque burden, increased arterial stiffness, and higher rates of CV events [88]. Notably, CT-derived indices of perivascular fat attenuation appear to capture dynamic PVAT remodeling and provide incremental prognostic information beyond traditional risk factors [89,90,91,92]. Viewed together with epicardial adipose tissue, PVAT thus emerges as both a mechanistic driver and an appealing therapeutic target in obesity-related vascular disease.

## 6. Adipose Remodeling After Weight Loss: Incomplete Reversal and Depot-Specific Memory

Beyond residual fibrosis and shifts in cellular composition, convergent multi-omic evidence supports a distinct, and only partially reversible, remodeling of adipose gene-regulatory architecture after obesity—such that weight loss can reduce adipocyte hypertrophy and improve systemic metabolic indices without fully re-establishing depot-level immunometabolic homeostasis. In mature adipocytes, prior obesity is associated with durable alterations in DNA methylation and histone landscapes, together with persistent changes in chromatin accessibility at enhancer and promoter elements governing lipid trafficking, mitochondrial energetics, proteostatic stress responses, and inflammatory signaling [93]. These regulatory regions may remain transcriptionally poised after weight normalization, resulting in incomplete reversion of obesity-induced expression programs and an exaggerated response to subsequent metabolic stressors, a pattern that is consistent with the clinical propensity for rapid weight regain and recurrent fibro-inflammatory activation. Importantly, this imprint is unlikely to be confined to adipocytes. Long-lived stromal and adipose progenitor populations can retain obesity-imposed regulatory states that shape the phenotype of newly generated adipocytes, while the post-obesity microenvironment—characterized by TGFβ-driven extracellular matrix remodeling, senescence-associated secretory signaling, and persistent organelle stress—may further stabilize inflammatory set-points despite fat mass reduction [94]. In parallel, a history of obesity may program innate immune compartments in a trained-immunity—like manner, whereby lipid- and TLR4-dependent cues remodel chromatin at AP-1—enriched inflammatory loci and license heightened cytokine responsiveness during secondary challenge or weight regain, providing a mechanistic substrate for inflammatory relapse with weight cycling [95,96].

Over the past two years, human adipose single-cell and single-nucleus atlases have provided compelling evidence that obesity drives depot-specific and cell type-specific remodeling of adipose tissue, and that this reconfiguration remains only partially reversible even after substantial weight loss (Table 1). A 2024 single-nucleus transcriptomic atlas of paired human subcutaneous and VAT provided a particularly illustrative reference point [97]. Employing snRNA-seq on five matched SAT–VAT pairs, supplemented by five additional VAT samples, Lazarescu and colleagues profiled 37,879 high-quality SAT nuclei and 83,731 VAT nuclei obtained from adults with body mass index (BMI) values ranging from 23.8 to 44.1 kg/m^2^ [97]. This comprehensive dataset delineated not only classical adipocytes characterized by ADIPOQ^+^AQP7^+^PLIN1^+^ expression but also several non-classical adipocyte subpopulations with distinct transcriptional and functional signatures [97]. They identified seven SAT clusters (SA1–7) and eight VAT clusters (VA1–8), with SAT dominated by one large classical population (85% of SAT adipocytes) and VAT by an analogous but slightly more metabolically stressed population (75% of VAT adipocytes) [97]. Importantly, VAT adipocytes were selectively enriched for pathways linked to IR (adjusted *p* = 0.007), adipocytokine signaling and inflammatory mediator regulation, supporting the clinical observation that visceral fat carries a higher cardiometabolic burden than SAT [97]. This atlas was further integrated with bulk RNA-seq from 73 patients with extreme obesity (preoperative BMI ≈ 54.5 ± 9.3 kg/m^2^), confirming by deconvolution that VAT in severe obesity remains transcriptionally skewed toward these inflammation-prone adipocyte states [97].

A 2024 study by Hinte and colleagues provided the clearest human evidence for an obesogenic memory [93]. They analyzed omental and subcutaneous biopsies from three independent bariatric cohorts—MTSS (Metabolic Tissue Single-cell Study; lean n = 5, obesity n = 8 before surgery, n = 8 two years after surgery), LTSS (Long-Term Surgery Study; lean n = 5, obesity n = 5 before, n = 5 after), and NEFA (Non-Esterified Fatty Acid Study; lean n = 8, obesity n = 7 before, n = 7 after)—and included only patients who had lost at least 25% of their baseline BMI at two years [93]. Across these datasets, they profiled 22,742 nuclei from omental AT and 15,347 nuclei from subcutaneous AT and showed that, despite near normalization of BMI and clinical indices, adipocytes retained a sizeable fraction of the obesity-induced transcriptional program [93]. In both omental and subcutaneous fat, metabolic genes that were downregulated in obesity—IGF1, LPIN1, IDH1, PDE3A in omentum; IGF1, DUSP1, GPX3 and GLUL in subcutaneous fat—remained downregulated two years after surgery, while gene-set enrichment demonstrated persistent upregulation of TGFβ-linked fibrotic pathways and apoptosis-related programs [93]. In parallel mouse experiments (48,046 nuclei across seven diet/weight-loss conditions), they showed that lipid-associated macrophages and non-perivascular macrophages that expand during high-fat feeding are not fully cleared after weight normalization; macrophages, adipocyte progenitors, endothelial cells and mature adipocytes all harbored retained differentially expressed genes, many of them pointing to lysosomal stress, ER stress and impaired fatty-acid oxidation [93]. Together, these data establish that weight loss reverses adipocyte size and improves systemic metabolism, but does not overwrite the obesity-imprinted transcriptional and epigenetic state of adipocytes, which likely underlies the well-described tendency toward rapid weight regain [94,98].

Subsequent 2025 commentaries and follow-up analyses have generalized this finding beyond adipocytes, showing that stromal, endothelial and immune compartments also exhibit lagging recovery [99,100]. In particular, multi-omics work building on the Nature dataset linked the persistence of DNA-methylation changes and chromatin-accessibility signatures to future transcriptional misregulation on re-exposure to an obesogenic diet—essentially an epigenetic blueprint that accelerates rebound adiposity and inflammatory activation [99,100]. Clinically oriented reviews framed this as a tissue-level analogue of metabolic memory: adipose tissue that has once been obese behaves differently from adipose tissue that has never been obese, even when contemporary BMI and glycaemia are matched [99,100].

When considered alongside the 2025 consensus statement from the Human Cell Atlas adipose bionetwork, these atlases gain interpretive cohesion [101]. That document introduced, for the first time, a standardized cell-type lexicon, adipocyte-competent single-nucleus RNA-sequencing (snRNA-seq) workflows, and benchmarking panels to enable rigorous mouse–human integration [101]. Led by Anne Loft, the working group argued that, without such harmonization, it is not possible to determine whether a persistent inflammatory progenitor or fibro-inflammatory progenitor reported after weight loss in one study corresponds to the lipid-associated macrophage (LAM/TREM2^+^), fibro-adipogenic, or endothelial-activated populations identified in another [101]. Accordingly, the roadmap recommends: (i) mandatory reporting of depot (SAT, VAT, EAT) and clinical context; (ii) explicit inclusion of adipocytes rather than limiting analyses to the stromal vascular fraction; (iii) parallel spatial profiling when anatomical adjacency is biologically consequential (e.g., EAT–atrium); and (iv) longitudinal sampling in interventional studies, directly applicable to trials of glucagon-like peptide-1 receptor agonists (GLP-1RAs) or bariatric surgery [101]. Collectively, these standards convert existing atlases into quantitative reference frameworks against which post-weight-loss states can be scored as fully reversed, partially reversed, or memory-persistent.

**Table 1:** Single-cell and multi-omic evidence of depot-specific adipose remodeling and persistent memory after weight loss.

Author, Year	Depot	Model/Population	Cellular Focus	Persistent Features Post-Weight Loss/Disease Regression	Key Findings
Lazarescu et al., 2025 [97]	Subcutaneous (SAT) and Visceral (VAT)	Human paired SAT–VAT samples (n = 5 pairs + 5 additional VAT); adults undergoing elective abdominal surgery; BMI 23.8–44.1 kg/m^2^; 80% female, 20% male	Single-nucleus transcriptomic profiling of 121,610 nuclei; identification of classical (lipid-metabolic) and nonclassical (angiogenic, immune, ECM, mito-ribosomal) adipocyte subtypes; pseudotime analysis of ASPC → adipocyte differentiation	Nonclassical adipocytes persist as intermediate states between progenitors and classical adipocytes, indicating partial reversibility of depot remodeling. VAT retains higher inflammatory and mito-ribosomal signatures; communication networks (leptin, IL-16) remain more pro-inflammatory than SAT even after metabolic improvement.	Defined 7 SAT (SA1–7) and 8 VAT (VA1–8) adipocyte clusters; classical adipocytes dominate but nonclassical subpopulations (immune-, angiogenic-, ECM-related) are conserved across datasets; depot-specific regulons (STAT4 vs. STAT6, PRRX1, MEF2C) govern fibrosis/inflammation; trajectory analysis shows that classical adipocytes emerge via functional loss from nonclassical states, implying adipocyte memory of prior inflammatory/fibrotic programming
Hinte et al., 2024 [93]	Human scAT and omental/visceral AT; mouse epididymal (epiAT), inguinal (ingAT), and BAT	Humans: lean vs. obese before bariatric surgery (T0) and 2 yr post-surgery (T1; ≥25% BMI loss) across MTSS, LTSS, NEFA cohorts. Mice: diet-induced obesity → chow-based weight loss → HFD re-challenge; AdipoERCre × NuTRAP for adipocyte-specific multi-omics; 80% female, 20% male	snRNA-seq across major AT cell types (adipocytes, APCs, endothelium, immune); in mice, adipocyte TRAP-seq, ATAC-seq, CUT&Tag (H3K4me3, H3K27me3, H3K4me1, H3K27ac); macrophage subtyping (LAM, PVM/NPVM)	In humans, many DEGs at obesity persist at 2 yr post-WL—especially in adipocytes/APCs/endothelium—with metabolic genes (e.g., IGF1, LPIN1, IDH1) remaining downregulated and fibrosis/apoptosis/TGFβ programs up. In mice, adipocyte promoters/enhancers retain memory (persistent H3K4me3/H3K27ac at inflammatory/ECM genes such as ICAM1, LYZ2, TYROBP; repressive H3K27me3 at adipogenesis/identity genes such as GPAM, ACACB, CYP2E1); LAM enrichment not fully normalized.	Demonstrates a durable “obesogenic” transcriptional and epigenetic memory in adipocytes after WL. Thousands of enhancers remain altered; 57–75% of persistent translational changes are accounted for by promoter/enhancer marks. Memory functionally primes adipocytes/mice for accelerated weight regain and inflammatory remodeling upon HFD re-challenge.
Cottam et al., 2022 [102]	Mouse epididymal WAT (eAT) ± sAT, liver (context)	Male C57BL/6J mice; diet-induced obesity → 9-wk weight loss (WL) → weight cycling (WC); CITE-seq with cell hashing; 33,322 immune cells across lean/obese/WL/WC	Single-cell transcriptomes + surface epitopes (CITE-seq) of immune compartment (T/NK/B cells, DCs, monocytes, macrophages incl. LAMs); RNA-velocity; MacSpectrum	Obesity-imprinted immune states persist after WL and intensify with WC: T-cell exhaustion (PD-1/TIGIT module) remains; Tregs stay low with reduced Il1rl1 (ST2); DCs retain mature/activated signatures (Ccr7_hi_/Fscn1_hi_/Cd274_hi_); classical monocytes keep lipid-handling/activation genes (Trem2, Cd36, Cd9, Cd81/63/86)	LAMs increase with obesity and do not normalize with WL; TRMs fall and remain low; activated DCs and exhausted CD8^+^ TEM persist through WL and worsen with WC, paralleling glucose intolerance independent of total fat mass—defining an immune memory of obesity that primes maladaptive responses on regain.
Miranda et al., 2025 [94]	Abdominal subcutaneous AT (primary focus; spatially resolved mapping of the adipose niche)	Human cohorts including men and women with extreme obesity undergoing therapeutic WL surgery (paired pre-/post-WL; n ≈ 25 pairs) and healthy lean controls (n ≈ 24); single-nucleus RNA-seq integrated with spatial transcriptomics (spatial cohorts n ≈ 4/group); 70% female, 30% male	Spatially resolved single-nucleus atlas profiling adipocytes, adipocyte stem/progenitor and stromal compartments, vascular cells (endothelial/mural), and immune populations (notably macrophage/monocyte lineages), coupled with regulatory network and metabolic flux inference.	In the immune compartment, WL reduces obesity-associated macrophage infiltration and inflammatory programs but does not fully reverse macrophage activation/metabolic priming, leaving persistent primed states despite substantial WL.	(i) Selective vulnerability to cellular stress/senescence in metabolic, precursor, and vascular cell states in obesity, with potent reversal after WL (marked depletion of p21+ senescent cells). (ii) WL reduces adipocyte hypertrophy and mechanotransduction/ECM constraint signatures and induces broad adipocyte metabolic activation (including lipid cycling and improved substrate handling), plausibly linking local remodeling to systemic metabolic benefit. (iii) Obesity-driven myeloid remodeling is only partially normalized—cell numbers decline and inflammatory polarization improves, yet activation persists.
Emont et al., 2025 [103]	Epididymal (EPI; visceral) and inguinal (ING; subcutaneous) WAT	Diet-induced obese male C57BL/6J mice on 60% HFD (8 weeks) undergoing vertical sleeve gastrectomy (VSG) vs. sham, or sham + semaglutide (0.04 mg/kg SC daily starting post-op day 5). Depots harvested at 10 days and ~25–35 days post-op; sNuc-seq performed on weight-matched animals (3 per cohort/timepoint), yielding 47,180 high-confidence nuclei.	Single-nucleus transcriptomics across adipose compartments (adipocytes; ASPCs; mesothelium in EPI; vascular; immune). Integrative analyses included subclustering, pseudobulk differential expression, pathway enrichment, and inferred cell–cell communication (CellChat), with emphasis on adipocyte state shifts and immune remodeling across weight-loss modalities.	Demonstrates incomplete molecular reset after weight loss: adipocyte programs segregate into (i) weight-dependent genes that revert toward lean, (ii) “weight memory” genes/pathways that remain obesity-imprinted despite improved metabolism, and (iii) semaglutide-specific transcriptional responses (i.e., altered uniquely by drug rather than obesity/weight per se). Metabolic improvement could occur before major immune-composition changes, underscoring potential dissociation between glycemic recovery and inflammatory-cell persistence/lag.	(1) VSG and semaglutide improved fasting glucose (with lower insulin trends), while immune–stromal remodeling became more apparent at later timepoints. (2) VSG elicited a stronger pro-inflammatory macrophage program in EPI, whereas semaglutide showed comparatively attenuated inflammatory remodeling. (3) Semaglutide induced depot-specific ASPC shifts (EPI vs. ING), consistent with divergent adipogenic trajectories. (4) Weight loss reduced obesity/stress-associated adipocyte states and, with semaglutide, favored a more beige/thermogenic signal in ING. (5) Despite weight loss, adipocytes remained distinct from lean controls due to persistent “weight memory” transcriptional programs.
Wang et al., 2025 [104]	BAT + beige adipose (inguinal beige; interscapular BAT)	Male C57BL/6J mice fed HFD for 16 weeks; randomized to Sham vs. SG at 20 weeks; tissues harvested 4 weeks post-op (24 weeks). snRNA-seq: 4 samples (Sham BAT, Sham Beige, SG BAT, SG Beige), 34,772 nuclei; adipose from 3 mice pooled per sequencing sample. Also re-analysis of a public human visceral adipose snRNA-seq dataset (GSE295708) comparing obese vs. weight-loss individuals.	snRNA-seq mapping of adipose microenvironment; emphasis on adipocytes (AP) and their interaction with fibroblastoid cells (FBO) and endothelial cells (EC); CellChat ligand–receptor signaling; GSVA (IGF/VEGF pathway activity); pseudotime/trajectory analysis of AP → FBO transition (AP subclusters incl. AP6); multiplex IF validation.	At 4 weeks post-SG, thermogenic depots show a shift toward a fibro-proliferative microenvironment: expansion of Nebl/Mylk-high “FBO” cells with concomitant reduction in AP and EC proportions; strengthened AP–FBO crosstalk dominated by IGF1–IGF1R signaling with suppression of VEGF-pathway activity in EC; emergence/enrichment of an AP6 subpopulation proximal to FBO in pseudotime, enriched for fibrosis-associated genes and an 11-gene putative transition regulator program (incl. Nucb2). (Persistence here is post-surgical at the analyzed timepoint, not a long-term follow-up claim.)	Bariatric surgery remodeled BAT/beige adipose toward a fibro-proliferative state, with strengthened AP–FBO crosstalk (dominant IGF1–IGF1R) and weakened AP–EC signaling (suppressed VEGF pathways). Trajectory analysis supported an AP → FBO continuum (AP6 near the terminus) and nominated 11 putative regulators (including NUCB2) coordinating this transition. IF confirmed fewer Emcn+ ECs, more Nebl+ FBOs, reduced Vegfa signal in ECs, and increased Igf1r in FBOs; cross-species re-analysis similarly showed reduced EC VEGF activity and higher NUCB2 in adipocytes after weight loss.

Abbreviations: SAT, subcutaneous adipose tissue; VAT, visceral adipose tissue; scAT, human subcutaneous adipose tissue; omental/visceral AT, omental (visceral) adipose tissue; eAT/eWAT, epididymal white adipose tissue (mouse, visceral); sAT, mouse subcutaneous adipose tissue; ingAT/iWAT, inguinal white adipose tissue (mouse, subcutaneous); BAT, brown adipose tissue; EAT, epicardial adipose tissue; ASPC, adipose stem/progenitor cell; APC(s), adipose progenitor cell(s) (not antigen-presenting cells); DC(s), dendritic cell(s); NK, natural killer (cell); TRM, tissue-resident macrophage; TEM, effector memory T cell; LAM(s), lipid-associated macrophage(s); PVM/NPVM, peri-/non-perivascular macrophage; snRNA-seq, single-nucleus RNA sequencing; CITE-seq, Cellular Indexing of Transcriptomes and Epitopes by sequencing; TRAP-seq, Translating Ribosome Affinity Purification sequencing; ATAC-seq, Assay for Transposase-Accessible Chromatin using sequencing; CUT&Tag, Cleavage Under Targets & Tagmentation; RNA-velocity, inference of transcriptional dynamics from spliced/unspliced RNA; DEG(s), differentially expressed gene(s); regulon, genes regulated by a common transcription factor; H3K4me3, active promoter mark; H3K27me3, repressive Polycomb mark; H3K4me1, primed enhancer mark; H3K27ac, active enhancer/promoter mark; ECM, extracellular matrix; TGFβ, transforming growth factor-beta; mito-ribosomal, mitochondrial/ribosomal program; BMI, body mass index; WL, weight loss; WC, weight cycling; HFD, high-fat diet; PD-1, programmed cell death protein-1; TIGIT, T-cell immunoreceptor with Ig and ITIM domains; AF, atrial fibrillation; Akt, protein kinase B; AMPK, AMP-activated protein kinase; ANXA1/AnxA1, Annexin A1; Arg1, arginase-1; AT1R, angiotensin II type-1 receptor; ATM, adipose tissue macrophage; β-arrestin, beta-arrestin; BP, blood pressure; cAMP, cyclic adenosine monophosphate; CD206, mannose receptor; ChemR23, chemerin receptor 23; CMKLR1, chemokine-like receptor 1; DRV1/GPR32, D-series resolvin receptor 1 (G-protein-coupled receptor 32); DRV2/GPR18, D-series resolvin receptor 2 (G-protein-coupled receptor 18); eNOS, endothelial nitric oxide synthase; ERK1/2, extracellular signal-regulated kinases 1/2; FFAR4/GPR120, free fatty acid receptor 4; FPR1, formyl peptide receptor 1; FPR2/ALX, formyl peptide receptor 2/lipoxin A4 receptor; Gαq/11, G alpha q/11; Gi/o, G alpha i/o; GLP-1, glucagon-like peptide-1; I/R, ischemia–reperfusion; JNK, c-Jun N-terminal kinase; LV, left ventricle/left-ventricular; LVEF, left-ventricular ejection fraction; Ly6C^high, Ly6C-high monocyte/macrophage subset; MAPK, mitogen-activated protein kinase; MDSC, myeloid-derived suppressor cell; NF-κB, nuclear factor kappa-B; PK, pharmacokinetics; RhoA, Ras homolog family member A; RyR2, ryanodine receptor 2; RvD1/RvD2, resolvin D1/resolvin D2; RvE1, resolvin E1; SREBP-1c, sterol regulatory element—binding protein-1c; STAT1, signal transducer and activator of transcription 1; TAB1, TAK1-binding protein 1; TAK1, TGF-β—activated kinase 1; TG, triglyceride; UCP1, uncoupling protein 1; FS, fractional shortening; Ca^2+^, calcium ion.

A cardio-adjacent layer of evidence comes from high-resolution atlases of epicardial and pericoronary fat. Liu et al. [105] profiled 73,386 nuclei from pericoronary EAT taken from 24 patients—8 controls, 8 with severe CAD, and 8 with CAD plus T2D—and identified 15 major clusters including adipocytes, multiple stromal/progenitor populations, and a rich immune compartment. CAD and CAD+T2D were characterized by expansion of CD83^high^ macrophages and FOSB^high^ adipocytes, along with a dysregulated secretome and circadian programs that are not seen in non-CAD EAT [105]. Because EAT is anatomically contiguous with the atrial and ventricular myocardium, these disease-imprinted EAT states provide a plausible route by which an adipose memory could perpetuate local inflammation and electrical vulnerability long after systemic risk factors are controlled [105].

This line of reasoning is reinforced by a 2024 study of 153 surgical patients, which showed that EAT in AF is enriched for CD69^+^PD-1^+^ tissue-resident memory (T_RM_) T cells and that spatial transcriptomics localises the most inflammatory, pro-fibrotic signalling to the narrow border between EAT and atrial myocardium [80]. After propensity matching, patients with AF still had higher T_RM_ frequencies (26 sinus-rhythm vs. 18 AF biological replicates), reduced clonal diversity and a shift toward KLRG1^+^, highly cytotoxic T_RM_ subsets capable of altering atrial calcium flux in vitro [80]. This is, in essence, an immunologic memory depot abutting the atrium, and it is unlikely to be erased merely by weight reduction, because T_RM_ cells are intrinsically long-lived and maintained by local cues rather than systemic adiposity alone [80].

Taken together, the accumulated multi-omic datasets converge on the concept that human adipose tissue can lose mass yet retain its molecular memory of obesity. Even following ≥25% BMI reduction and two years of sustained follow-up, omental and subcutaneous adipocytes preserve a discernible transcriptional signature reflective of prior metabolic stress, macrophage populations remain polarized toward lipid-associated macrophage (LAM)-like phenotypes, and cardio-proximal depots such as EAT maintain pro-inflammatory, memory-enriched immune niches. This residual program—now extensively charted across SAT, VAT, and EAT, as well as across both surgical and pharmacological weight-loss interventions—has progressed beyond descriptive observation and should be regarded as a therapeutically actionable substrate rather than a mere biological vestige of obesity.

From a mechanistic standpoint, these observations can be systematised along three interrelated axes: depot, cellular compartment, and functional consequence. Visceral and cardio-proximal depots (VAT, EAT) consistently emerge as loci of persistent inflammatory, fibrotic, and stress-related activity; adipocytes, stromal cells, endothelial cells, and tissue-resident immune subsets each retain discrete components of an obesogenic imprint; and, at the functional level, this imprint appears to sustain heightened cardiometabolic and arrhythmic susceptibility despite apparent clinical improvement. Within this framework, weight reduction alone predominantly decreases adipose mass without fully resetting the underlying immunometabolic circuitry, thereby highlighting the rationale for therapies that actively remodel this memory. This perspective provides a direct conceptual link to resolution pharmacology, in which specialised pro-resolving mediators and allied pathways are harnessed to reprogramme adipose and immune niches towards durable, cardioprotective remodelling rather than transient debulking of fat.

## 7. Resolution Pharmacology Against Obesity-Related Inflammation to Prevent CV Disease

**Figure 3:**
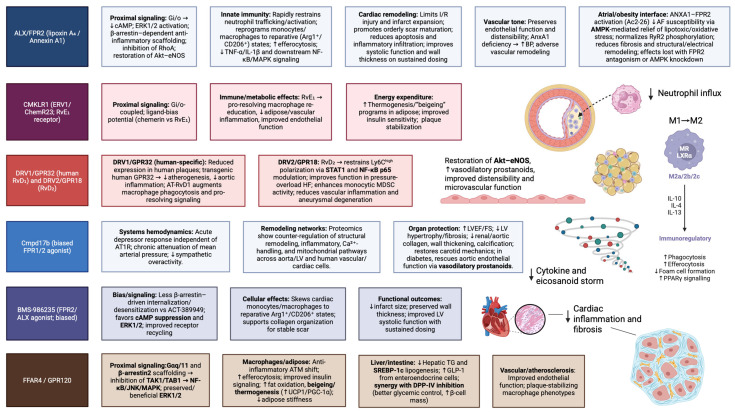
Comparative signaling and outcomes of pro-resolving G protein-coupled receptor targets in obesity-related inflammation for CV disease prevention. Obesity-associated CMD is sustained by chronic, non-resolving inflammation; emerging strategies aim not only to block pro-inflammatory initiation pathways, but to pharmacologically engage endogenous resolution programs via specialized pro-resolving mediator (SPM) receptors. ALX/FPR2 signaling, activated by Annexin A1 and lipoxin A_4_ or by synthetic FPR2-biased agonists, limits leukocyte trafficking, promotes reparative macrophage phenotypes, preserves endothelial function, reduces infarct size, and lowers atrial fibrillation susceptibility in obesity through AMPK- and Akt–eNOS—dependent pathways. CMKLR1 (ERV1/ChemR23) activation by resolvin E_1_ re-educates adipose macrophages, improves insulin sensitivity, and augments thermogenesis while reducing vascular inflammation, supporting biased agonists that reproduce pro-resolving signaling while avoiding chemerin-like pro-inflammatory effects. D-series resolvin receptors DRV1/GPR32 and DRV2/GPR18 constrain vascular inflammation, attenuate adverse cardiac remodeling, promote myeloid pro-resolving programs, and show translational potential through epimer-stabilized analogs such as 17R-RvD_2_, whereas biased agonists, including BMS-986235 and Cmpd17b link FPR-family signaling to improved post-infarct remodeling, lower blood pressure, and protection from hypertensive vascular injury. FFAR4/GPR120 agonism further couples β-arrestin/Gαq signaling to suppression of NF-κB/MAPK activity, macrophage polarization, hepatic lipid unloading, GLP-1—dependent metabolic improvement, and potential reduction of epicardial/visceral adipose inflammation relevant to AF and HFpEF risk.

The next generation of anti-inflammatory, metabolo-protective therapies for obesity-associated inflammation is likely to arise from pharmacologic engagement of the resolution circuitry rather than exclusive suppression of initiation pathways [106]. Specialized pro-resolving mediators (SPMs)—notably lipoxins, maresins and resolvins—actively terminate inflammation and restore tissue homeostasis through cognate G-protein-coupled receptors expressed on adipocytes, macrophages, and CV cell types, with RvD1/RvD2 directly correcting adipose inflammation and IR in diet-induced obesity (Figure 3 [107,108]. Ex vivo human data further support these vascular actions, with DHA-derived SPMs (RvD1, RvD5, MaR1) dampening vasoconstriction and inflammatory cytokine release in human saphenous vein segments [109]. Selective receptor agonism or biased-ligand strategies at these targets offer a means to potentiate endogenous resolution programs while minimizing broad immunosuppression [107]. The following section synthesizes receptor-level targets with the most compelling mechanistic depth and translational traction. 

### 7.1. ALX/FPR2 (Lipoxin A_4_/ANXA1 Receptor)

Annexin A1 (AnxA1)—an endogenous agonist of the FPR2/ALX receptor—exerts cardiometabolic protection across acute and chronic settings. In models of myocardial ischemia/reperfusion and permanent coronary occlusion, AnxA1 limits injury, an effect in acute phases attributable to restrained leukocyte trafficking and activation [110,111]. With sustained administration, AnxA1 reprograms the cardiac immune milieu, promoting a reparative macrophage phenotype [110]. In experimental type 2 diabetes, AnxA1 mitigates metabolic derangements and secondary microvascular pathology through inhibition of RhoA and restoration of Akt–eNOS signaling [112,113,114]. Independent preclinical work in inflammatory arthritis demonstrates that AnxA1 both prevents and reverses diastolic dysfunction, hypertrophy, and myocardial fibrosis, coincident with suppression of pro-inflammatory cytokines and pro-fibrotic mediators, reduced fibroblast and activated T-cell infiltration, and expansion of anti-fibrotic MHC-II^low cardiac macrophages [115]. Complementing these gain-of-function data, AnxA1 deficiency is associated with elevated blood pressure throughout life and age-accentuated vascular remodeling, impaired distensibility, and endothelial dysfunction [116,117].

Concordant vascular effects are seen with LXA_4_ signaling through FPR2/ALX: in ApoE^−^/^−^ mice, aspirin-triggered LXA_4_ reduces atherosclerotic burden, macrophage infiltration, and increases fibrous-cap collagen content, supporting a direct plaque-stabilising role of pro-resolving FPR2 activation [118]. Selectively engaging FPR2/ALX may confer advantages over dual FPR1/FPR2 agonism in CV settings. Although one study implicated FPR1 in cardiomyocyte survival with preservation of left-ventricular function after ischemia–reperfusion [119], other reports describe the opposite direction: genetic or pharmacologic FPR1 inhibition reduced cardiomyocyte apoptosis, neutrophil infiltration, and inflammatory signaling, culminating in improved post-ischemic remodeling [120,121]. Beyond injury models, FPR1 blockade lowered arterial pressure in Dahl salt-sensitive rats maintained on a low-salt diet [122]. A recent synthesis underscores multiple detrimental contributions of FPR1 to CV disease progression [123].

FPR2-selective peptidomimetics derived from Annexin A1—CR-AnxA1_2–50_ and CR-AnxA1_2–48_—have each conferred robust protection against acute myocardial ischemia–reperfusion (I/R) injury in murine models [124,125]. Extending these observations, a solid-phase—synthesized, FPR2-preferential agonist, RTP-026, was recently shown to couple cardioprotection with immunomodulation in rat I/R paradigms [126]. In a 2-h reperfusion model, RTP-026 administered at the onset of reperfusion reduced infarct size by ~50%, with maximal effect at 50 μg/kg, accompanied by curtailed recruitment and activation of neutrophils and classical monocytes within the injured myocardium. Systemically, RTP-026 tempered vascular inflammatory tone, lowering circulating TNF-α, IL-1β, KC, PGE_2_, and PGF_2_α. In a 24-h reperfusion protocol, intravenous dosing at 0, 3, and 6 h (30 μg/kg each) yielded an approximate 40% reduction in necrotic myocardial area [126].

Adding to this mechanistic framework, a recent integrative bioinformatic and experimental study identified FPR2 as a hub gene jointly up-regulated in atrial tissue from patients with AF and in obesity, and demonstrated that activating the ANXA1–FPR2 axis with the ANXA1 mimetic Ac2-26 reduces AF susceptibility in high fat diet obese mice by alleviating atrial lipotoxic stress via AMPK signaling [127]. In vivo, FPR2 antagonism increased AF inducibility and fibrosis, whereas Ac2-26 improved insulin resistance, mitigated atrial lipid accumulation and oxidative stress, normalized RyR2 phosphorylation, and limited structural/electrical remodeling; cardiomyocyte-targeted AMPK knockdown abrogated these benefits [127]. Complementary cell studies showed that Ac2-26 attenuates palmitate-induced apoptosis and oxidative injury and restores fatty-acid oxidation (↑PPARα, ↑CPT1B, ↑p-ACC), effects reversed by FPR2 blockade or AMPK inhibition [127].

### 7.2. ERV1/ChemR23 (CMKLR1; RvE_1_ Receptor)

Recent structure-guided insights into chemerin–CMKLR1 recognition and signaling open the door to ligand-bias engineering, while metabolic studies position CMKLR1 as a node that couples immune re-education to energy expenditure via adipose thermogenesis and beigeing [128]. In obese states, engagement of CMKLR1 by RvE_1_ improves insulin sensitivity and augments thermogenic programming, changes that are directionally aligned with reduced adipose and vascular inflammation, improved endothelial function, and potentially greater plaque stability [129,130], as RvE1 directly attenuates atherosclerotic lesion growth and inflammation in ApoE^−^/^−^ models, including in combination with statin therapy [131]. By contrast, chemerin—often elevated in obesity and linked to adverse vascular phenotypes—can reinforce chemotactic and pro-inflammatory tone [132]. These ligand-divergent effects support a medicinal-chemistry strategy of biased CMKLR1 agonism that preferentially recapitulates RvE_1_-like, pro-resolving signaling while minimizing chemerin-driven responses, thereby targeting cardiometabolic risk at the interface of adipose biology, vascular homeostasis, and atherogenesis [130,133].

### 7.3. DRV1/GPR32 (Human RvD_1_ Receptor) and DRV2/GPR18 (RvD_2_ Receptor)

DRV1/GPR32 and DRV2/GPR18 anchor the D-series resolvin axis with direct relevance to cardiometabolic inflammation. Although GPR32 lacks a murine ortholog, human tissues and humanized models demonstrate that DRV1 signaling constrains vascular inflammation and atherogenesis: GPR32 expression is reduced in human atherosclerotic plaques, and transgenic expression of human GPR32 confers smaller lesions, diminished aortic inflammation, and heightened macrophage pro-resolving responses; AT-RvD1 further augments phagocytosis and intracellular signaling in this context [134].

For DRV2/GPR18, convergent evidence across hematopoietic and CV models indicates that RvD_2_–GPR18 activation reprograms innate immunity and limits adverse remodeling: in pressure-overload heart failure, the RvD_2_/GPR18 axis improves function and attenuates Ly6C^high^ macrophage polarization via STAT1 and NF-κB p65 pathways, with genetic loss of GPR18 abolishing benefit [135]; in abdominal aortic aneurysm, RvD_2_/GPR18 signaling enhances monocytic MDSC function, reduces vascular inflammation, and mitigates structural degeneration [136]. Clinically oriented chemistry and formulation advances are beginning to address pharmacokinetic liabilities of native mediators; notably, the recently elucidated 17R-epimer of RvD_2_ displays potent pro-resolving actions at picomolar–nanomolar concentrations and activates the RvD_2_ receptor, strengthening the translational rationale for long-acting or epimer-stabilized D-series agents [137]. In atheroprone mice, systemic MaR1 or RvD2 administration similarly rebalanced lesional lipid mediator profiles, halted atheroprogression, reduced necrotic core formation and macrophage accumulation, and thickened fibrous caps, directly linking D-series resolvins and maresins to plaque stabilization [138].

Translational readouts for SPM–G-protein-coupled receptors (GPCR) programs in obesity-linked CVD should pair molecular and tissue-level indices with clinical physiology. Practical early end points include targeted LC-MS/MS panels of circulating SPMs and precursors (for example, DHA- and EPA-derived mediators such as 17-HDHA/18-HEPE), which are quantifiable in human biofluids and have been applied to identify D-series mediators including 17R-RvD_2_ [137]. At the tissue level, profiling adipose macrophage states—particularly LAM/TREM2^+^ populations implicated in lipid-stress remodeling—can index resolution competence within obese adipose depots [139]. For cardiometabolic interfaces, depot-specific imaging (e.g., EAT characterization) and rhythm metrics (e.g., AF burden where relevant) can be co-registered with immune and lipid-mediator signatures to capture target engagement and downstream CV effects.

### 7.4. BMS-986235

BMS-986235—an FPR2/ALX agonist—demonstrated cardioprotection in murine and rat models of myocardial infarction induced by permanent LAD ligation, reducing infarct size, preserving left-ventricular systolic function, and attenuating adverse remodeling [140]. Mechanistically, treatment increased macrophage arginase-1 transcripts and CD206 expression, alongside greater collagen deposition within the infarct zone—features consistent with a transition toward a reparative, pro-resolution macrophage phenotype that supports orderly scar maturation under FPR2/ALX engagement [141].

Lupisella et al. [140] contrasted the molecular pharmacology of BMS-986235 with the clinically studied FPR2/ALX agonist ACT-389949, focusing on post-receptor signaling and desensitization dynamics. ACT-389949 drives prolonged receptor internalization with sustained desensitization [142], exhibits lower potency for cAMP inhibition, and shows greater efficacy in β-arrestin recruitment relative to BMS-986235. Notably, agonist-induced FPR2 recycling to the plasma membrane was observed with BMS-986235 but not with ACT-389949. In short-term in vivo dosing after myocardial infarction, both ligands skewed cardiac monocytes/macrophages toward a pro-resolving phenotype; however, with longer dosing, only BMS-986235 preserved infarct wall thickness and improved left-ventricular systolic function [140]. Subsequent work corroborates a signaling bias of BMS-986235 (and compound 43) away from β-arrestin recruitment and receptor-trafficking pathways, and toward cAMP suppression and ERK1/2 phosphorylation—pathways classically linked to cardioprotection [143,144]. Molecular docking further suggests distinct FPR2 interaction fingerprints: WKYMVm and ACT-389949 share key contact residues that differ from those engaged by BMS-986235 and compound 43 [143].

### 7.5. Cmpd17b

Extending these observations beyond ischemic remodeling, a biased small-molecule FPR1/2 agonist (compound 17b; Cmpd17b) has also been shown to mitigate hypertension-driven CV injury in an angiotensin II—induced model [145]. In this setting, Cmpd17b attenuated the chronic rise in mean arterial pressure, suppressed sympathetic overactivity, and elicited an acute depressor response that was independent of AT1 receptor signaling [145]. Functionally, treatment improved left-ventricular ejection fraction and fractional shortening, reduced LV hypertrophy and interstitial fibrosis, and limited renal and aortic collagen accumulation, vascular wall thickening, mucin/fibrin deposition, and calcification, with partial restoration of carotid distensibility and strain [145]. At a systems level, an extensive proteomic analysis encompassing approximately 6000 proteins in thoracic aorta, left ventricle, and human vascular and cardiac cell models showed that Cmpd17b broadly opposed hypertension-driven alterations in pathways related to structural remodeling, inflammatory signalling, calcium handling, and mitochondrial function [145]. Importantly, part of the Cmpd17b-responsive proteome overlapped with dysregulated protein signatures identified in human datasets of aortic remodeling and hypertrophic left ventricle, indicating that the experimentally modulated networks map onto clinically relevant disease pathways [145].

In a complementary streptozotocin-induced diabetes model, eight weeks of Cmpd17b treatment restored aortic endothelial function, an effect linked to the induction of vasodilatory prostanoid pathways [146]. Taken together, these observations support the concept that selectively biased FPR signalling can integrate blood pressure control with tissue-repair programmes, thereby complementing the FPR2-selective profile of BMS-986235 and reinforcing the rationale for resolution-oriented therapeutic strategies in hypertension-associated cardiac and vascular disease.

### 7.6. FFAR4/GPR120 Agonism

FFAR4 (GPR120) is a long-chain fatty-acid—sensing GPCR enriched in adipose tissue, macrophages, and enteroendocrine compartments. Agonist engagement of FFAR4 activates Gα_q/11_ and β-arrestin signaling, thereby tempering NF-κB/MAPK activity, shifting macrophages toward anti-inflammatory phenotypes, and improving insulin action in obese models. In keeping with this mechanism, selective small-molecule agonists show complementary benefits: cpdA enhances glucose tolerance and insulin sensitivity and alleviates steatosis while suppressing macrophage inflammatory signaling in a GPR120-dependent fashion, whereas TUG-891 rapidly increases fat oxidation, promotes thermogenic/beige adipose activation, and lowers fat mass through mitochondrial pathways in brown adipocytes [147,148,149]. Extending these observations, FFAR4 stimulation reduces hepatic triglyceride burden and downregulates lipogenic programs in vivo, supporting broader cardiometabolic utility [147].

Translational momentum is underpinned by two complementary advances. First, combination therapy: in diet-induced obese mice, oral cpdA synergized with DPP-IV inhibition (sitagliptin) to further enhance glucose homeostasis, increase circulating GLP-1, reduce adiposity, and restore β-cell mass/proliferation—implicating a clinically exploitable gut–islet axis [150]. Second, discovery platforms: structure-informed, machine-learning—guided pipelines integrated with docking and molecular dynamics are yielding chemically tractable FFAR4 agonists with favorable interaction fingerprints and predicted stability, broadening the lead space beyond classical long-chain fatty-acid mimetics [151].

By curbing adipose and macrophage inflammatory tone and promoting thermogenic/beiging programs, FFAR4 activation offers a plausible route to reduce visceral/epicardial adipose inflammation and stiffness, with downstream benefits for vascular dysfunction and diastolic loading—pathways germane to AF/HFpEF risk modification. Evidence that FFAR4 agonism mitigates steatosis and modulates plaque-relevant macrophage phenotypes further aligns the receptor with atherometabolic risk reduction [147].

## 8. Limitations and Future Directions

Despite convergent mechanistic and translational signals, several constraints temper inference. First, the current knowledge base is unevenly distributed across models and populations. Much of the mechanistic insight into adipose memory and depot-specific remodelling stems from rodent models of diet-induced obesity, weight loss, and weight cycling, and from highly selected human cohorts—most notably bariatric-surgery populations with ≥25% BMI reduction, patients with advanced CAD undergoing cardiac surgery, or individuals with AF in whom EAT is sampled intraoperatively. Data on medically treated obesity, from patients with earlier or less extreme adiposity, from HFpEF or primary arrhythmic cohorts, and from under-represented groups (e.g., different ancestries, older adults, women with HFpEF/AF) remain sparse. As such, the generalisability of depot-specific and immune memory signatures to the broader population with obesity-associated CV disease is not yet established.

Second, many of the single-cell, spatial, and multi-omic datasets discussed in Section 6 are cross-sectional or involve relatively short to intermediate follow-up, and most focus on surgical weight loss. Longitudinal profiling under contemporary pharmacotherapies (GLP-1RAs, dual and triple agonists, SGLT2 inhibitors) or structured lifestyle programmes is only beginning to emerge. It remains unclear to what extent different non-pharmacological (dietary, exercise, bariatric) and pharmacological strategies differentially remodel adipocyte, stromal, endothelial, and immune compartments across SAT, VAT, and EAT, and whether specific intervention signatures translate into durable reductions in CV events, arrhythmia burden, or HFpEF progression. Robust, depot-resolved time-course studies that couple omics with imaging and clinical outcomes are therefore a priority.

Third, the resolution-pharmacology arm of the field remains predominantly preclinical. Most data on FPR2/ALX agonists (AnxA1 mimetics, BMS-986235, Cmpd17b), resolvin receptors (DRV1/GPR32, DRV2/GPR18), CMKLR1, and FFAR4 derive from small-animal models of myocardial ischaemia–reperfusion, pressure overload, Ang II—induced hypertension, streptozotocin diabetes, or diet-induced obesity. Human evidence is largely confined to ex vivo studies, limited biomarker work, or indirect inferences (e.g., receptor expression in atherosclerotic plaques, overlap between modulated proteomic networks and human disease signatures). Interspecies differences further complicate translation—for instance, the absence of a murine ortholog for DRV1/GPR32 necessitates humanised or chimeric models, which may not fully recapitulate human cardiometabolic disease.

Fourth, pharmacology at pro-resolving GPCRs is heterogeneous. Within the formyl-peptide receptor family, ligand- and receptor-bias phenomena introduce variability in post-receptor trafficking, desensitisation, and downstream signalling. Head-to-head preclinical work already demonstrates that FPR2 agonists differ in β-arrestin recruitment, receptor recycling, and functional durability, complicating class effect assumptions and making dose selection for early-phase clinical trials non-trivial. Native SPMs have short half-lives, and although epimer-stabilised analogues (e.g., 17R-RvD2) exhibit potent pro-resolving actions, these compounds remain at an early developmental stage, with limited pharmacokinetic and safety data in humans. Proposed translational readouts—LC-MS/MS—based SPM panels, depot-specific adipose immune-state profiling (e.g., LAM/TREM2^+^ macrophages, EAT TRM subsets), and cardio-adjacent imaging metrics—are promising but not yet validated as surrogate endpoints or routinely standardised across platforms and centres.

Finally, many of the immunologic insights at the EAT–atrium interface are associative. Enrichment of TRM T-cell subsets and spatially localised pro-fibrotic signalling at the EAT–myocardium border in AF suggests plausible mechanisms by which adipose memory might sustain arrhythmic vulnerability, but functional causality, reversibility, and therapeutic modifiability in humans remain to be established, and confounding by comorbidities and concomitant therapies cannot be excluded.

Collectively, these limitations delineate a clear agenda for future work. For non-pharmacological strategies, there is a need for depot-resolved, longitudinal studies comparing lifestyle, bariatric, and pharmacological weight-loss interventions with respect to their capacity to erase—or at least attenuate—adipose and immune memory in VAT and EAT and to modify CV endpoints. For pharmacological strategies, carefully staged development of resolution-based agents will require small mechanistic trials that co-register lipid-mediator profiles, adipose and blood immune phenotypes, depot-specific imaging (including EAT), and arrhythmic or haemodynamic readouts. Only through such model- and population-aware programmes will it be possible to translate the conceptual promise of resolution pharmacology into rational, evidence-based interventions for obesity-related CV disease.

## 9. Conclusions

In summary, obesity-driven inflammation operates across systemic and depot-specific axes—most notably within epicardial adipose tissue—to promote electrophysiologic vulnerability, diastolic loading, and atherogenesis, while emerging single-cell atlases reveal that adipose memory persists despite substantial weight loss, predisposing to relapse of metabolic and CV risk. These convergent insights argue for a precision framework that integrates cardiometabolic therapies with resolution-oriented pharmacology targeting pro-resolving GPCRs, alongside standardized, depot-aware imaging and molecular end points to verify target engagement and capture mechanistic benefit. By coupling burden-reducing interventions with strategies that actively terminate inflammation and remodel the cardio-proximal adipose niche, the field can move beyond weight-centric paradigms toward durable modification of the immunometabolic substrate that underlies AF, HFpEF, and coronary disease. Realizing this vision will require harmonized phenotyping across SAT, VAT, and EAT, rigorous validation of lipid-mediator and immune signatures as decision tools, and randomized trials powered for hard CV outcomes, thereby translating biological plausibility into sustained clinical gains.

## Data Availability

No new data were created or analyzed in this study. Data sharing is not applicable to this article.

## Supplementary Materials

The following supporting information can be downloaded at: https://www.mdpi.com/article/10.3390/ijms27010535/s1.
