# Supplementary figures and images for: Inflammation and Resolution in Obesity-Related Cardiovascular Disease
Source: Int J Mol Sci. 2026 Jan 5;27(1):535. doi: 10.3390/ijms27010535 (PMC12786577; doi:10.3390/ijms27010535)

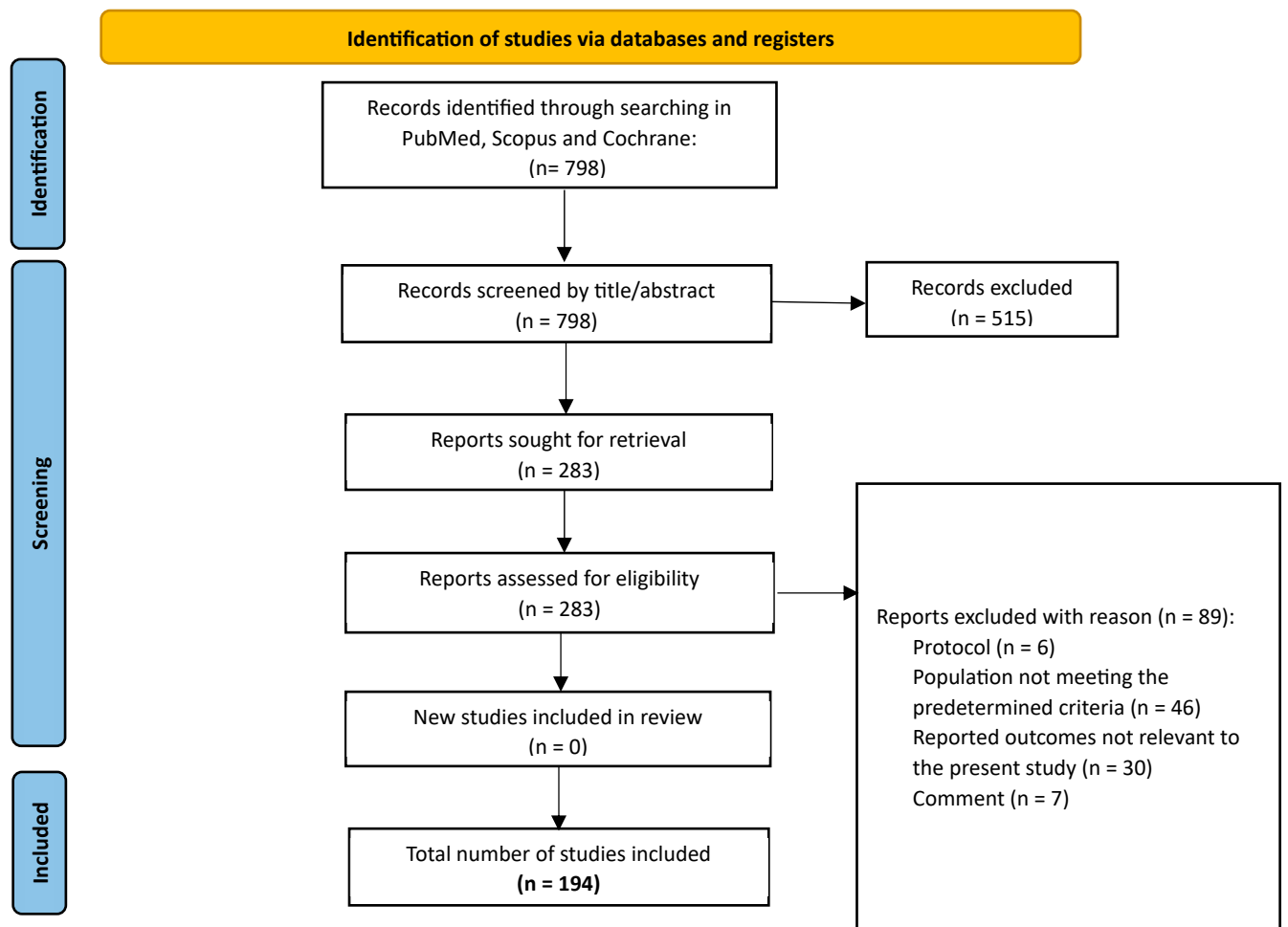

**Figure S1.** Flow diagram summarizing database searching and record screening.

Supplement: Supplementary file 1 [file ijms-27-00535-s001.zip › ijms-4061696-supplementary.pdf]
